# Supplementary material for: hMRP8-ATTAC Mice: A New Model for Conditional and Reversible Neutrophil Ablation
Source: Cells. 2022 Jul 30;11(15):2346. doi: 10.3390/cells11152346 (PMC9367557; doi:10.3390/cells11152346)
Supplement: Supplementary file 1 [file cells-11-02346-s001.zip › cells-1795984 supp figures.pdf]

Supplementary Figures

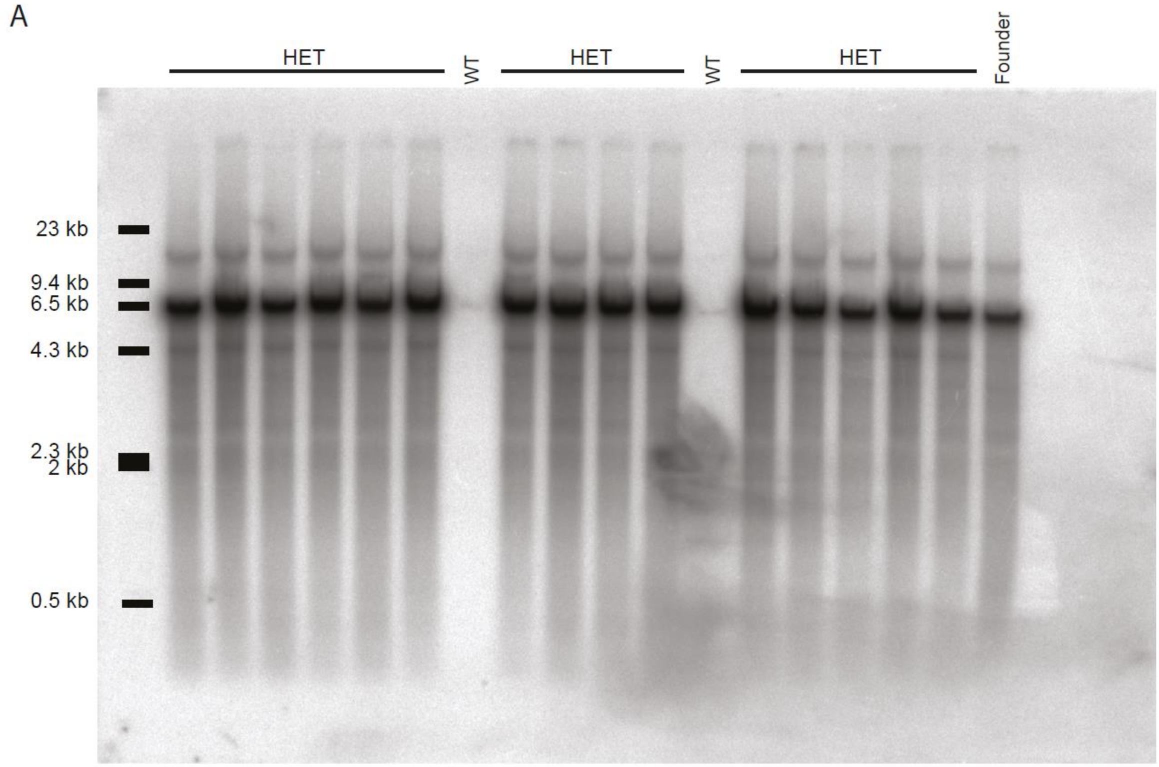

Supplementary Figure S1. The hMRP8-ATTAC construct is integrated in one site in multiple copies. (A) Southern Blot analysis of toe-derived genomic DNA of Founder 3, and heterozygous and WT F1 progeny. The transgene is identified by the hybridization of the GFP probe (concatamer band of 6114kb). HET, heterozygous. WT, wild type.

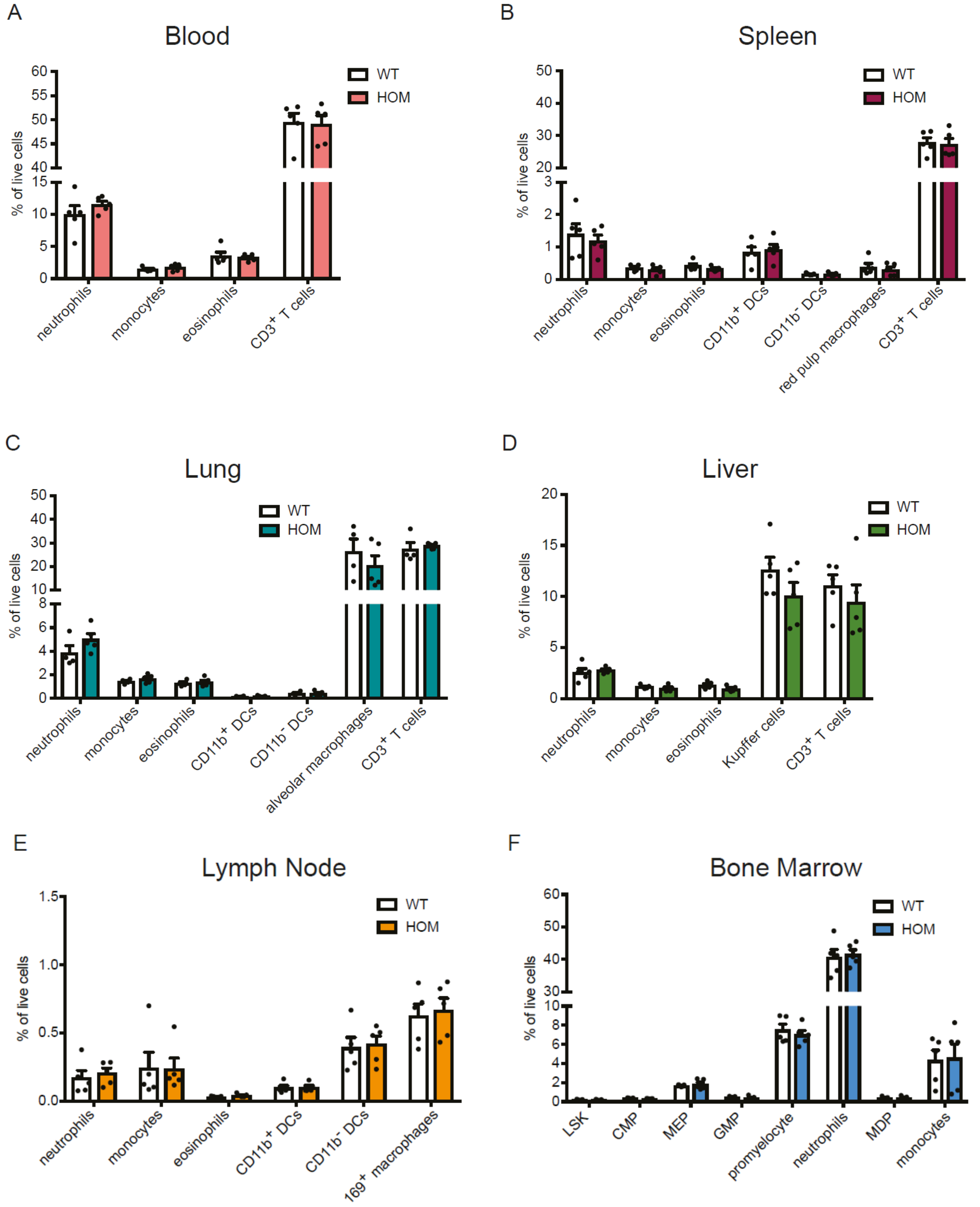

Supplementary Figure S2. No changes in the immune cell composition in hMRP8-ATTAC mice. (A-F) Immune cell proportions in the blood (A), spleen (B), lung (C), liver (D), lymph node (E) and bone marrow (F) of hMRP8-ATTAC mice and WT littermates (n=4-5). HOM, homozygous. Data are mean values ± SEM.

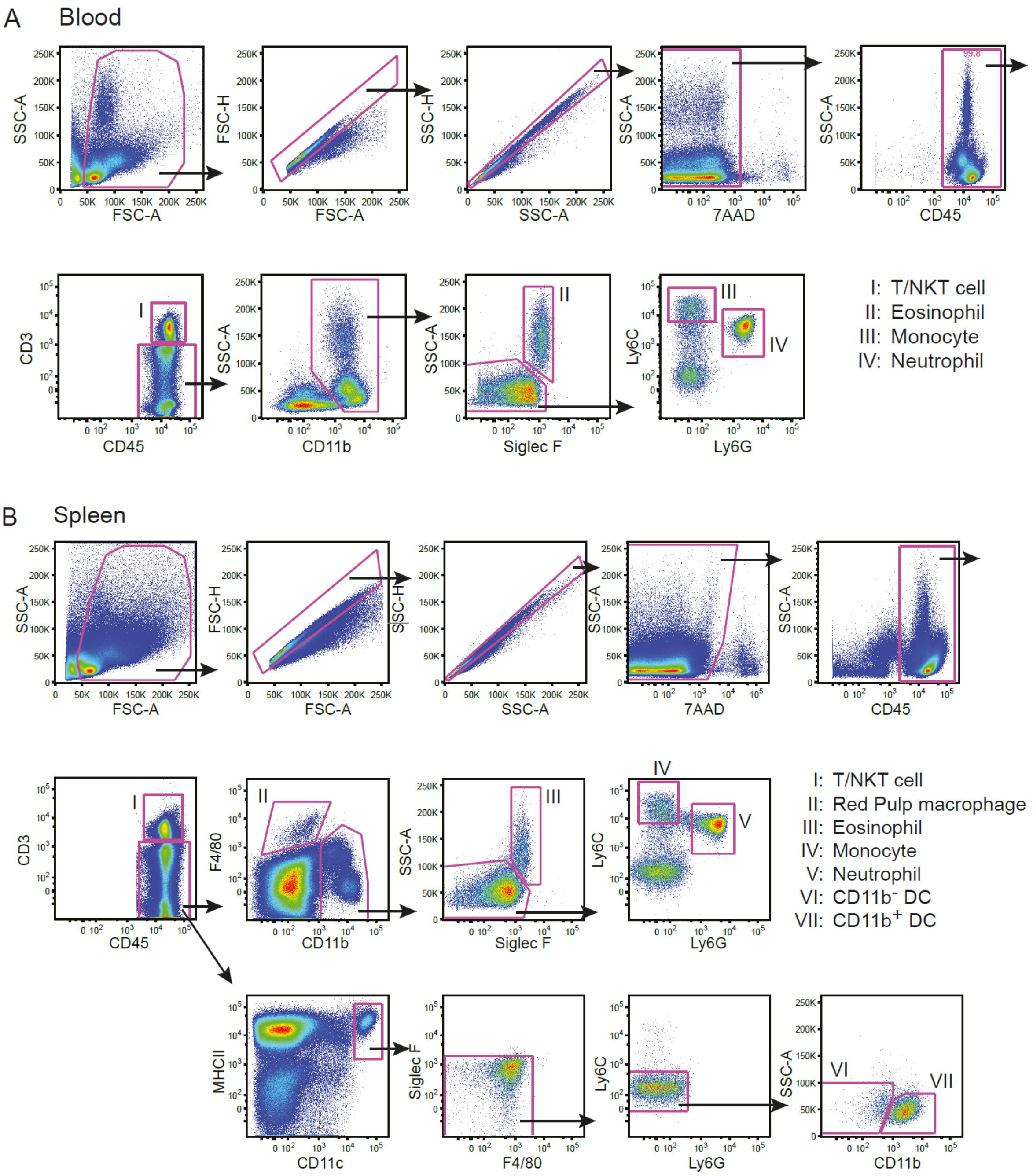

Supplementary Figure S3. Flow cytometry gating strategy for blood and spleen samples. (A-B) Representative dot plots of blood (A) and spleen (B) of a homozygous hMRP8-ATTAC mouse illustrating the gating strategy for the identification of immune cell populations. Antibody panel used: “Panel 1” (Suppl. Table 1). Arrows indicate directionality of sub-gates.

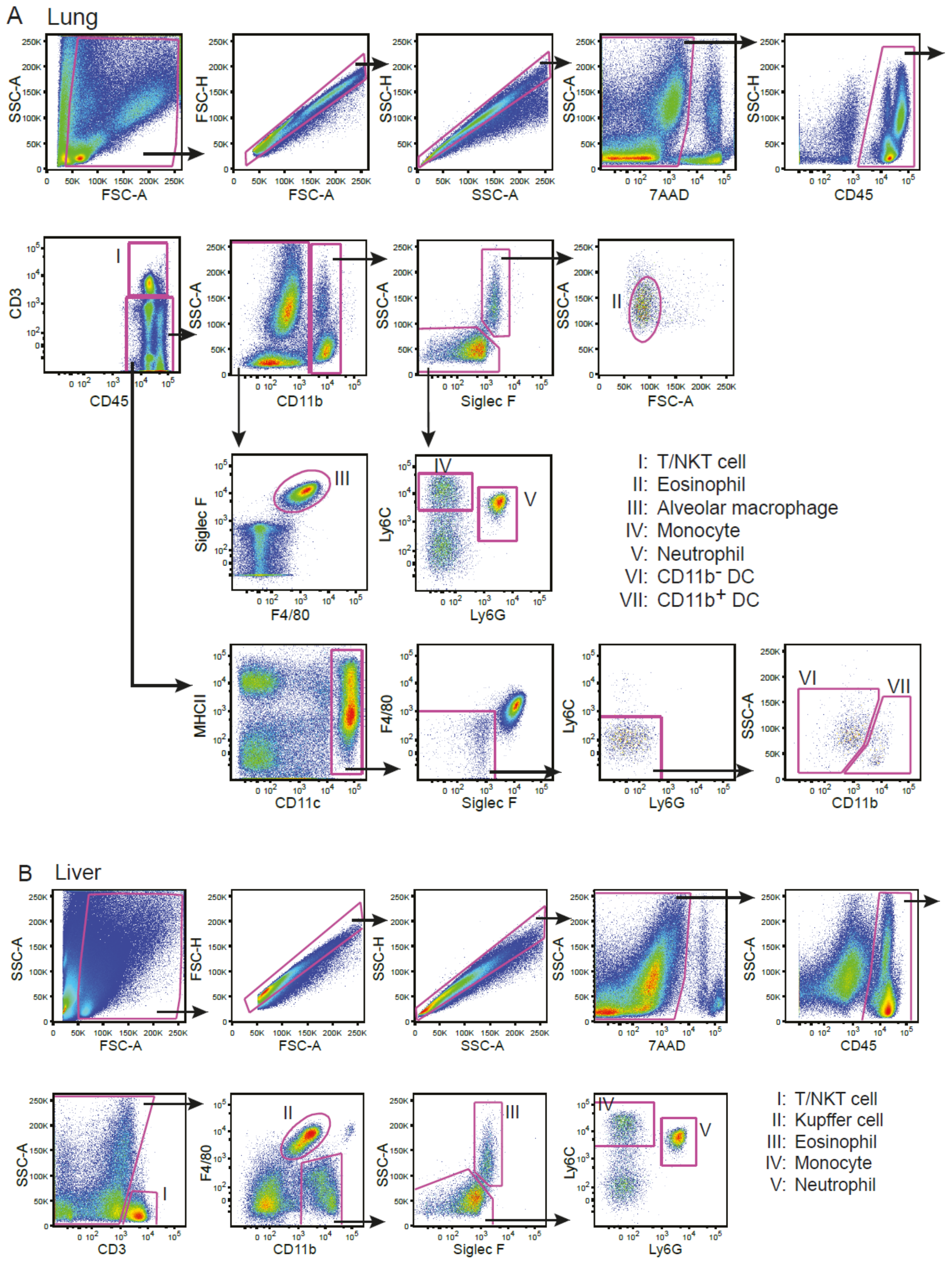

Supplementary Figure S4. Flow cytometry gating strategy for lung and liver samples. (A-B) Representative dot plots of lung (A) and liver (B) of a homozygous hMRP8-ATTAC mouse illustrating the gating strategy for the identification of immune cell populations. Antibody panel used: “Panel 1” (Suppl. Table 1). Arrows indicate directionality of sub-gates.

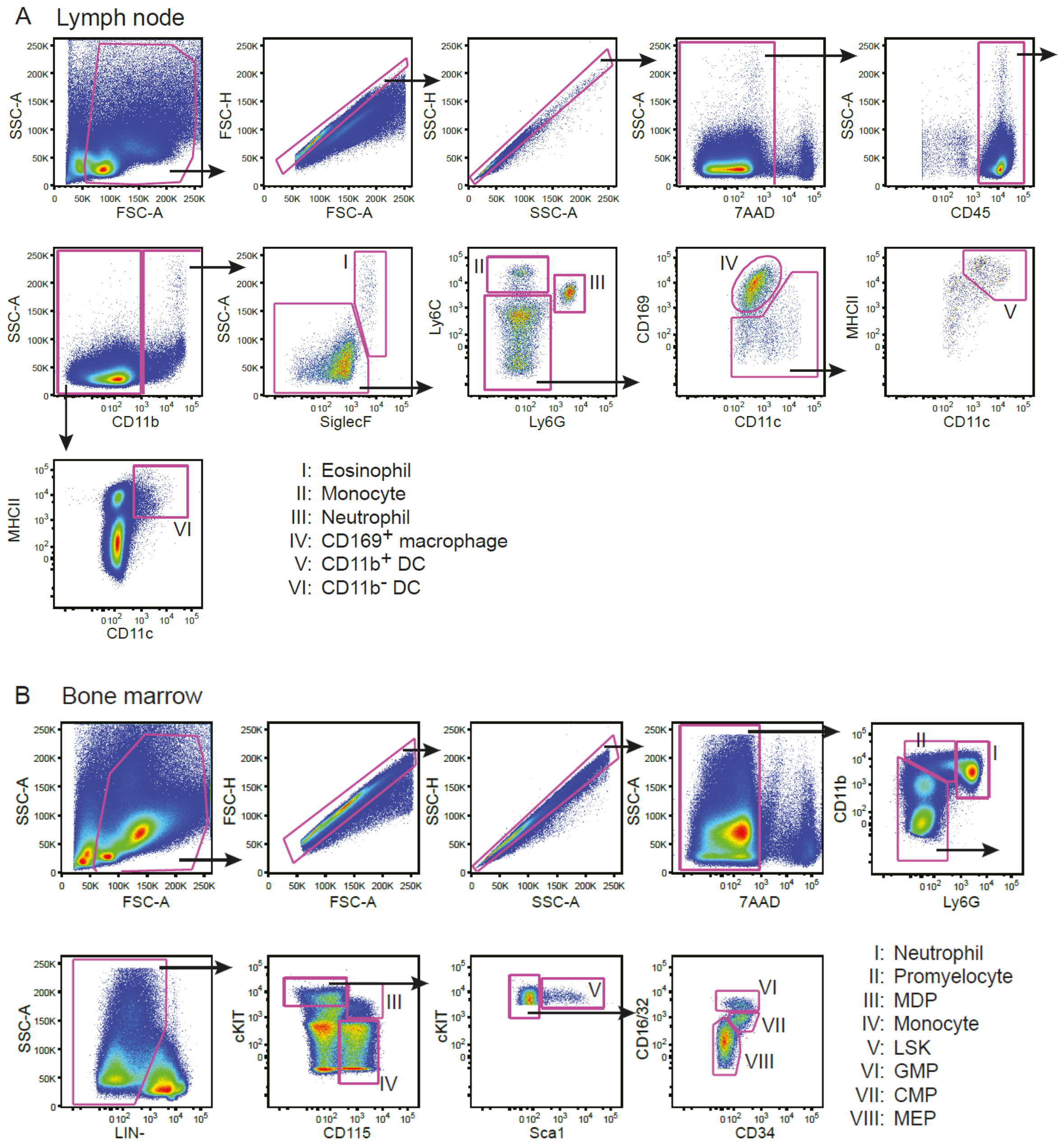

Supplementary Figure S5. Flow cytometry gating strategy for lymph node and bone marrow samples. (A-B) Representative dot plots of lymph node (A) and bone marrow (B) of a homozygous hMRP8-ATTAC mouse illustrating the gating strategy for the identification of immune cell populations. Antibody panel used: "Panel 2" for lymph node and "Panel 3" for bone marrow (Suppl. Table 1). Arrows indicate directionality of sub-gates.

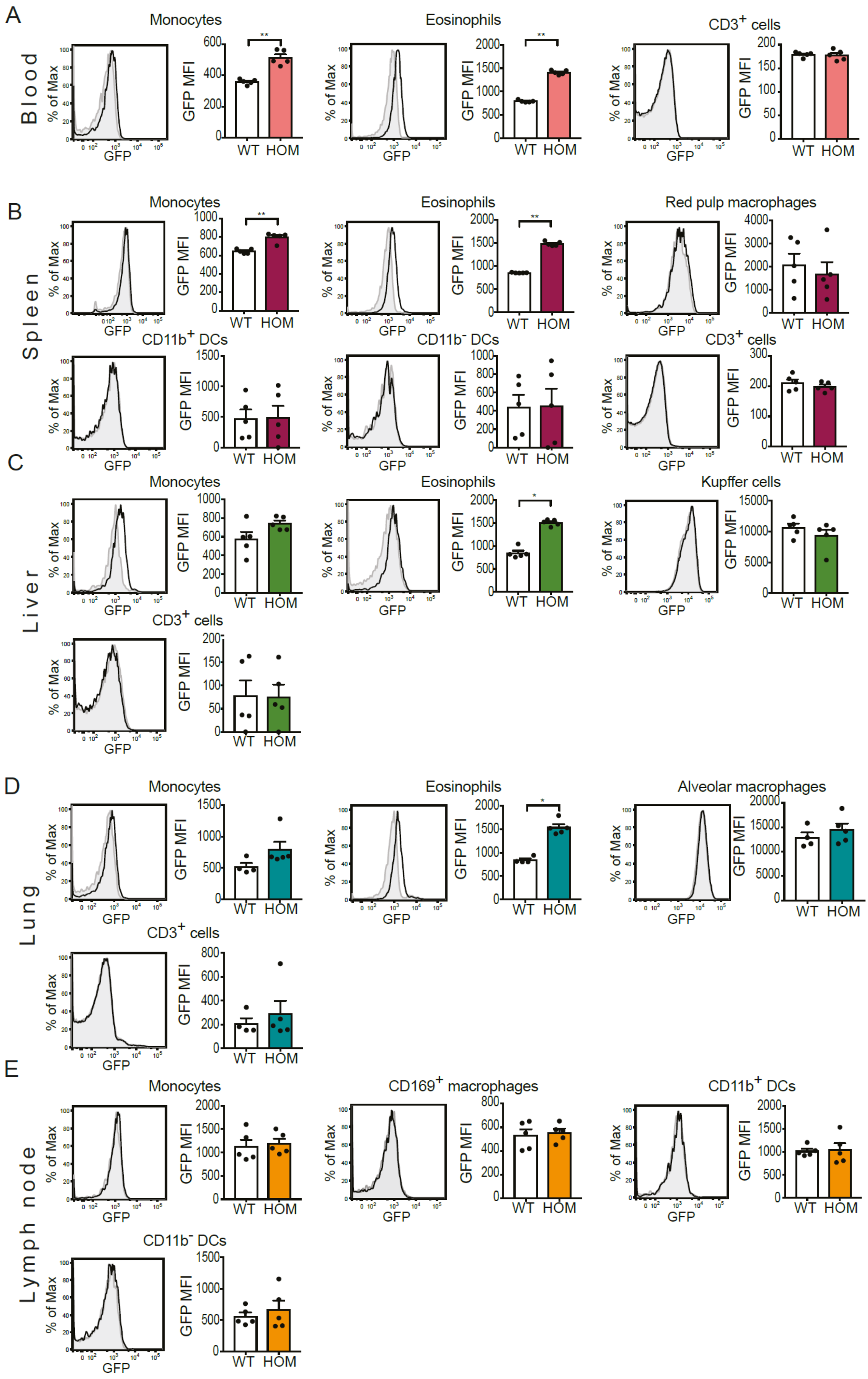
A
Blood
Monocytes
Eosinophils
CD3+ cells
% of Max
GFP
GFP MFI
WT HOM
B
Spleen
Monocytes
Eosinophils
Red pulp macrophages
CD11b+ DCs
CD11b- DCs
CD3+ cells
C
Liver
Monocytes
Eosinophils
Kupffer cells
CD3+ cells
D
Lung
Monocytes
Eosinophils
Alveolar macrophages
CD3+ cells
E
Lymph node
Monocytes
CD169+ macrophages
CD11b+ DCs
CD11b- DCs

Supplementary Figure S6. Monocytes and eosinophils in the hMRP8-ATTAC model express low levels of the transgene. (A-E) Representative histograms of GFP expression and quantification of the Median Fluorescence Intensity (MFI) of GFP in several immune cells from blood (A), spleen (B), liver (C), lung (D) and lymph node (E) of homozygous female hMRP8-ATTAC mice (n=5) compared to WT littermates (n=4-5), as determined by flow cytometry. WT, wild type; HOM, homozygous. Data are mean values ± SEM. *p<0.05, **p<0.01 by Mann–Whitney test.

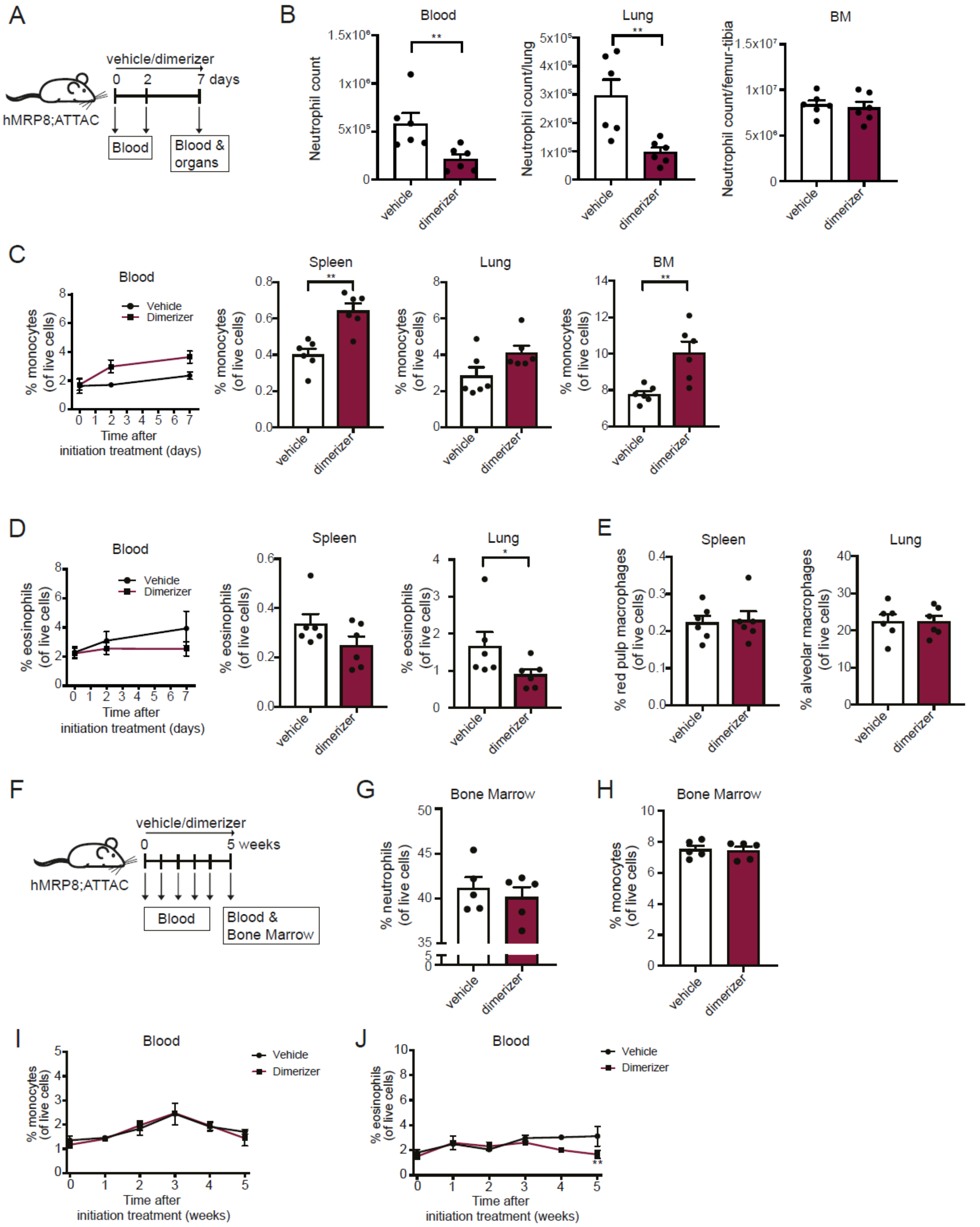

Supplementary Fig. S7 | No reduction in monocyte and eosinophil frequencies upon dimerizer treatment in hMRP8-ATTAC mice. (A) Experimental design of 1-week vehicle or dimerizer treatment in homozygous female hMRP8-ATTAC mice. (B) Total number of neutrophils in blood, lung and bone marrow after seven days of vehicle- or dimerizer treatment. Absolute neutrophil counts were calculated for whole blood, one lung and one femur-tibia as follows: (initial total cell number * frequency of total live neutrophils)/100. (C-E) Frequencies of monocytes (C), eosinophils (D) and tissue-resident macrophages (E) in blood over time (first panel) and in spleen, lung and bone marrow after seven days of treatment with vehicle or dimerizer in hMRP8-ATTAC mice, as determined by flow cytometry (n=6/group). (F) Schematic representation of experimental design of 5-week vehicle or dimerizer treatment in hMRP8-ATTAC mice (n=5/group). (G-H) Frequencies of neutrophils (G) and monocytes (H) in the bone marrow of hMRP8-ATTAC mice treated with vehicle or dimerizer for five weeks. (I-J) Circulating monocyte (I) and eosinophil (J) frequencies were determined by flow cytometry during five weeks of vehicle- or dimerizer treatment in hMRP8-ATTAC mice. Data are mean values ± SEM. *$p<0.05$, **$p<0.01$ by

Mann–Whitney test (B-D) or **p<0.001 by unpaired t-test followed by Holm-Sidak's correction for multiple comparisons (J).

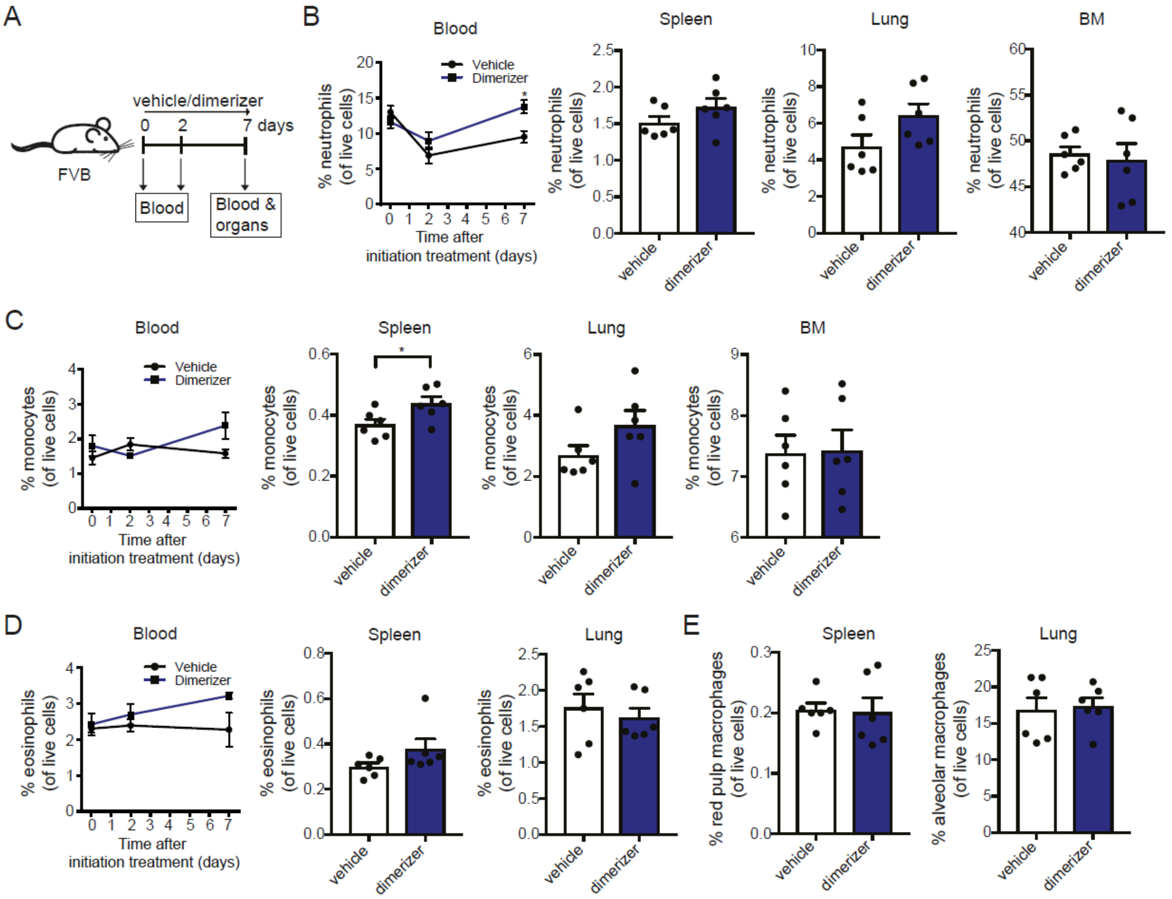

Supplementary Figure S8. Dimerizer treatment has modest effects on neutrophils in WT mice. (A) Schematic representation of 1-week treatment regime in WT mice. (B-D) Frequencies of neutrophils (B), monocytes (C) and eosinophils (D) in blood over time (first panel) and in spleen, lung and bone marrow after seven days of vehicle or dimerizer treatment in WT mice, as determined by flow cytometry (n=6/group). (E) Frequencies of resident macrophages in spleen and lung of WT mice treated with vehicle or dimerizer for seven consecutive days, as determined by flow cytometry (n=6/group). Data are mean values ± SEM. *p<0.05 by unpaired t-test followed by Holm-Sidak's correction for multiple comparisons (B) or *p<0.05 by Mann–Whitney test (C).

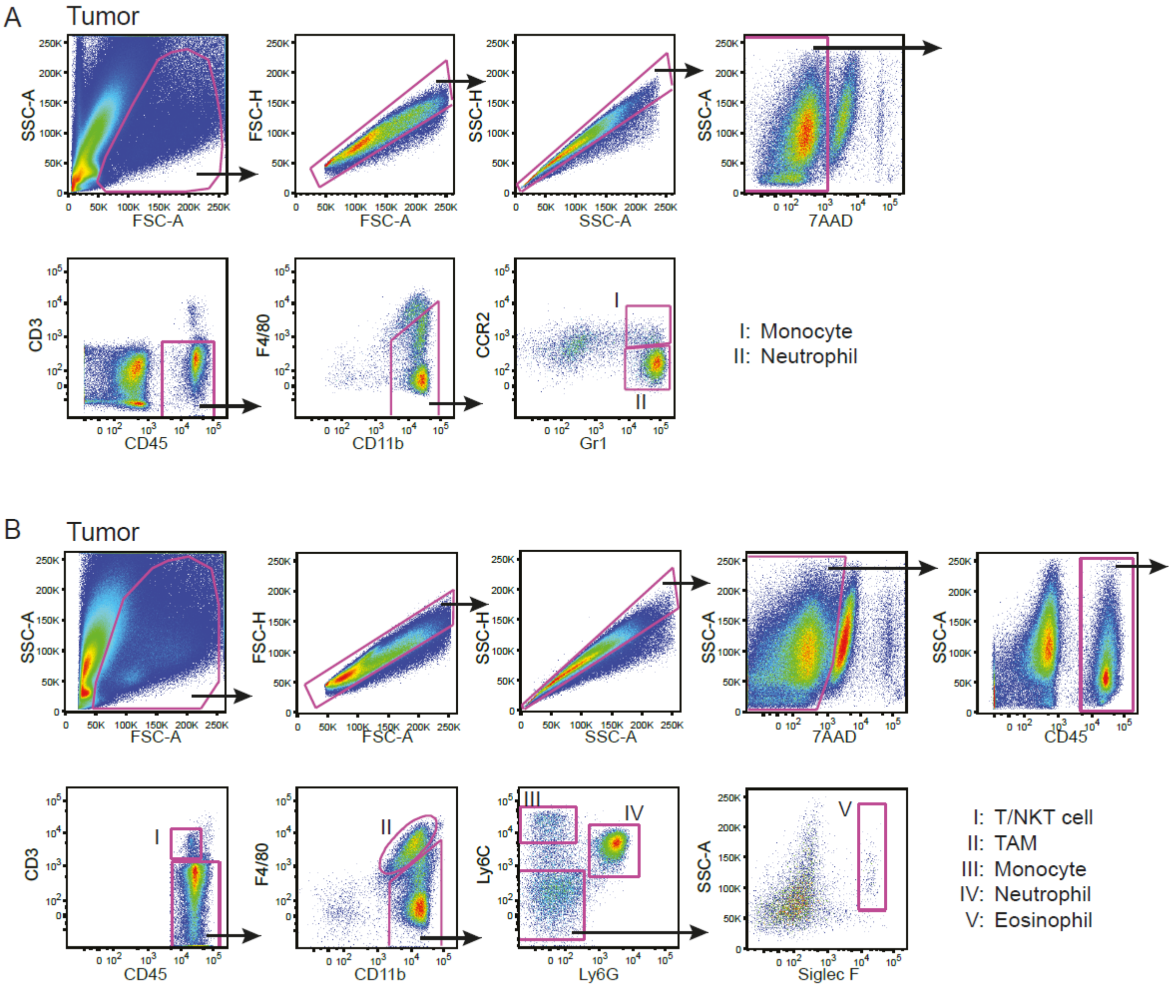

Supplementary Figure S9. Flow cytometry gating strategy for tumor samples. (A-B) Representative dot plots of tumor samples of a homozygous hMRP8-ATTAC mouse orthotopically transplanted with *K14-cre;Cdh1$^{F/F}$;Trp53$^{F/F}$* mammary tumor piece illustrating two gating strategies for the identification of cell populations. Antibody panel used: "Panel 4" in (A) and "Panel 5" in (B) (Suppl. Table 1). Arrows indicate directionality of sub-gates. Gating strategy of (A) was used for WEA tumor-bearing mice and both strategies (A-B) were used for KEP tumor-bearing mice.
