# Supplementary material for: hMRP8-ATTAC Mice: A New Model for Conditional and Reversible Neutrophil Ablation
Source: Cells. 2022 Jul 30;11(15):2346. doi: 10.3390/cells11152346 (PMC9367557; doi:10.3390/cells11152346)
Supplement: Supplementary file 1 [file cells-11-02346-s001.zip › cells-1795984 supp table.pdf]

Supplementary Table S1: List of antibodies used for flow cytometry analyses

Panel 1. Blood, spleen, lung and liver

Antibody	Fluorophore	Clone	Vendor	Dilution
CD45	BUV395	30-F11	BD Biosciences	1:200
CD3	PE-cy7	145-2c11	eBioscience	1:200
Siglec F	BV605	E50-2440	BD Biosciences	1:200
CD11b	BV786	M1/70	BD Biosciences	1:400
CD11c	PE	N418	eBioscience	1:200
Ly6G	AF700	1A8	Biolegend	1:200
Ly6C	Ef450	HK1.4	eBioscience	1:400
F4/80	APC	BM8	eBioscience	1:200
MHC class II	APCef780	M5/114.15.2	eBioscience	1:200

Panel 2. Lymph node

Antibody	Fluorophore	Clone	Vendor	Dilution
CD45	BUV395	30-F11	BD Biosciences	1:200
Siglec F	BV605	E50-2440	BD Biosciences	1:200
CD11b	BV786	M1/70	BD Biosciences	1:400
CD11c	BV421	HL3	BD Biosciences	1:200
Ly6G	AF700	1A8	Biolegend	1:200
Ly6C	PEcy7	HK1.4	Biolegend	1:400
F4/80	APC	BM8	eBioscience	1:200
MHC class II	APCef780	M5/114.15.2	eBioscience	1:200
CD169	PE	3D6.112	Biolegend	1:200

Panel 3. Bone marrow

Antibody	Fluorophore	Clone	Vendor	Dilution
CD11b	APCef780	M1/70	eBioscience	1:400
CD115	BV711	AFS98	Biolegend	1:200
Ly6G	AF700	1A8	Biolegend	1:200
Ter119	PE	TER-119	Biolegend	1:400
CD3	PE	145-2C11	Biolegend	1:400
CD4	PE	H129.19	Biolegend	1:400
CD8	PE	53-6.7	eBioscience	1:400
CD19	PE	eBio1D3	eBioscience	1:400
Sca1	BV421	D7	Biolegend	1:400
CD117	BV605	2B8	BD Biosciences	1:200
CD16/32	BUV395	2.4G2	BD Biosciences	1:200
CD34	AF647	RAM34	BD Biosciences	1:100

Panel 4. Dimerizer treatment in tumor-bearing hMRP8;ATTAC mice: blood, spleen, lung and tumor

Antibody	Fluorophore	Clone	Vendor	Dilution
CD45	BUV395	30-F11	BD Biosciences	1:200
CD3	PE-cy7	145-2c11	eBioscience	1:200
CD11b	BV786	M1/70	BD Biosciences	1:400
CCR2	PE	475301	R&D systems	1:100
Gr1	BV421	RB6-8C5	Biolegend	1:200
F4/80	APC	BM8	eBioscience	1:200

Panel 5. Dimerizer treatment in tumor-bearing hMRP8;ATTAC mice: blood, spleen, lung and tumor

Antibody	Fluorophore	Clone	Vendor	Dilution
CD45	BUV395	30-F11	BD Biosciences	1:200
CD3	PE-cy7	145-2c11	eBioscience	1:200
Siglec F	PE	E50-2440	BD Biosciences	1:200
CD11b	BV786	M1/70	BD Biosciences	1:400
CD11c	BUV737	HL3	BD Biosciences	1:100
CD117	BV605	2B8	BD Biosciences	1:100
Ly6G	AF700	1A8	Biolegend	1:200
Ly6C	Ef450	HK1.4	eBioscience	1:400
F4/80	APC	BM8	eBioscience	1:200
MHC class II	APCef780	M5/114.15.2	eBioscience	1:200
